# Walking on a Tissue-Specific Disease-Protein-Complex Heterogeneous Network for the Discovery of Disease-Related Protein Complexes

**DOI:** 10.1155/2013/732650

**Published:** 2013-12-28

**Authors:** Thibault Jacquemin, Rui Jiang

**Affiliations:** MOE Key Laboratory of Bioinformatics and Bioinformatics Division, TNLIST/Department of Automation, Tsinghua University, FIT 1-107, Beijing 100084, China

## Abstract

Besides the pinpointing of individual disease-related genes, associating protein complexes to human inherited diseases is also of great importance, because a biological function usually arises from the cooperative behaviour of multiple proteins in a protein complex. Moreover, knowledge about disease-related protein complexes could also enhance the inference of disease genes and pathogenic genetic variants. Here, we have designed a computational systems biology approach to systematically analyse potential relationships between diseases and protein complexes. First, we construct a heterogeneous network which is composed of a disease-disease similarity layer, a tissue-specific protein-protein interaction layer, and a protein complex membership layer. Then, we propose a random walk model on this disease-protein-complex network for identifying protein complexes that are related to a query disease. With a series of leave-one-out cross-validation experiments, we show that our method not only possesses high performance but also demonstrates robustness regarding the parameters and the network structure. We further predict a landscape of associations between human diseases and protein complexes. This landscape can be used to facilitate the inference of disease genes, thereby benefiting studies on pathology of diseases.

## 1. Introduction

With a vast amount of genetic variants detected by such techniques as traditional genome-wide association studies [1, 2] and recent exome sequencing studies [3, 4], connecting functional implications of these genetic variants to human inherited diseases has now become a standard task [5]. For genetic variants occurring in protein coding regions, a typical approach to this task is to screen out a set of candidate genes around the genomic positions where the genetic variants occur and then prioritize the candidates to identify genes that are most likely to be associated with a disease of interest [6, 7].

To achieve this goal, quite a few approaches have been proposed from the perspective of computational systems biology. For example, Endeavour resorted to the guilt-by-association principle [8] to rank candidate genes according to their functional similarities to a set of predefined seed genes [9]. Cipher integrated a phenotype similarity profile and a protein-protein interaction (PPI) network to make a global inference of disease genes [10]. The idea of relying on phenotype similarities between diseases instead of between pre-defined seed genes to make inferences has then been extended by a series of methods, including RWRH [11], PRINCE [12], AlignPI [13], MAXIF [14], and many others [15–17]. In these studies, PPI networks have also been dominantly used to provide a simplified yet systematic measure of functional similarities between gene products [7], and recent studies have shown the advantage of using tissue-specific PPI networks over using generic ones [18–20].

However, a biological function usually arises from the cooperation of multiple proteins. These proteins link to each other by noncovalent interactions, forming a protein complex. Hence, genetic variants occurring at different loci might affect the structure of a member protein of a complex, alter the function of the entire complex, and cause a disease. For example, it has been reported that seven pathogenic genes responsible for a heterogeneous syndrome called Fanconi anemia (FA) form a protein complex with functions related to DNA repair [21]. Therefore, besides the prioritization of candidate genes for a disease of interest, it is also of great importance to identify protein complexes underlying a query disease, thereby shedding light on biological processes and functional mechanisms of the occurrence and development of the disease under investigation.

Some methods for identifying disease genes have paid attention to linking protein complexes to diseases and then made use of such information to facilitate the prediction of disease genes. For example, Lage et al. proposed to identify the aggregates of proteins connected to a candidate protein in a PPI network as a protein complex by a virtual pull-down procedure and infer the association between the candidate protein and a query disease based on members of the protein complex [15]. Vanunu et al. proposed to analyze the PPI network and to establish a prioritization procedure in order to identify densely connected subnetworks that contain high scoring proteins as disease-related protein complexes [12]. Yang et al. proposed to infer disease genes from relationship between protein complexes and diseases [22]. These studies demonstrate that association relationships between protein complexes and a query disease could enhance the inference of disease genes. However, so far it still lacks a computational approach to systematically analyze potential relationships between known protein complexes and human diseases.

With the above understandings, we propose in this paper a computational systems biology approach for the identification of protein complexes that are related to a query disease via a random walk model on a heterogeneous network that is composed of a disease-disease similarity layer, a tissue-specific protein-protein interaction layer, and a protein complex membership layer. Starting from the query disease at the disease layer, our method simulates the process in which a random walker travels in the three-layered disease-protein-complex network, scores a protein complex using the probability that the walker stays in the protein complex at the steady state, and then ranks candidate protein complexes according to their scores. With a series of large-scale leave-one-out cross-validation experiments, we systematically show that our method not only possesses high performance but also demonstrates robustness to parameters involved and the network structure. As an application of our approach, we predict a landscape of associations between human diseases and known protein complexes and provide free downloads of the prediction results at http://bioinfo.au.tsinghua.edu.cn/jianglab/complex.

## 2. Methods

### 2.1. Overview of the Proposed Method

**Figure 1:**
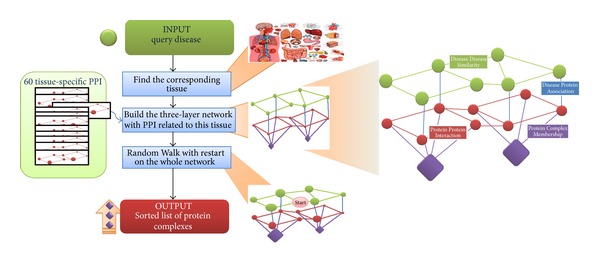
Illustration of the proposed method. Our method takes as inputs a query disease and a set of candidate protein complexes and gives a ranking list of the candidates as the output. For this purpose, we construct a tissue-specific disease-protein-complex heterogeneous network, apply a random walk with restart algorithm to the network to obtain scores for candidate protein complexes, and rank the candidates according to their scores.

We model the problem of identifying protein complexes associated with a query disease as a prioritization problem and propose to solve this problem with a three-step approach. As illustrated in Figure 1, given a query disease and a set of predefined protein complexes as inputs, we first identify the tissue to which the disease is most likely related. Then, we construct a tissue-specific disease-protein-complex heterogeneous network, which is composed of three layers: a disease-disease similarity layer on the top, a protein-protein interaction layer in the middle, and a protein complex membership layer at the bottom. In this procedure, we use a PPI network that is specific to the tissue identified in the first step as the middle layer. Finally, we apply a random walk with restart algorithm to the three-layer network to calculate a score for each candidate complex and further rank the candidates to obtain a ranking list as the output.

### 2.2. Construction of the Disease-Protein-Complex Network

The disease-protein-complex network is composed of three layers. The top layer is a disease-disease similarity network derived from a phenotype similarity profile [23]. The middle layer is a tissue-specific PPI network derived using generic PPI information [24] and tissue-specific gene expression data [25]. The bottom layer reflects relationships between proteins and complexes that are extracted from the database [26].

At the top layer, given a disease phenotype similarity profile (a real-valued matrix) that quantifies pairwise overlaps of diseases in their clinic traits, we construct the disease-disease similarity network by using two strategies. First, with a *k*-nearest neighbour (*k*-NN) strategy (used as the default in our study), we link each disease to its *k* nearest neighbours, which correspond to the *k* highest phenotype similarity scores. Second, with a *δ-*threshold strategy, we set up a cut-off value *δ* and then connect two diseases by an undirected edge if and only if their similarity is greater than or equal to the cut-off. In both strategies, we further consider two variations for edges: weighting edges by the original similarity values or treating edges as unweighted.

At the middle layer, given generic PPI network and tissue-specific gene expression data, we get a tissue-specific PPI network from the literature [18]. These networks have been constructed by using one of the two following strategies. The first one is a naïve node removal (NR) strategy: a tissue-specific network is constructed by removing proteins that are not expressed in the given tissue from the generic PPI network. The second one is an edge reweight (ERW) strategy (used as the default in our study): each edge in the tissue-specific network is assigned a weight (controlled by a parameter 0 ≤ rw ≤ 1 with default value 0.1 [18]), reflecting the possibility that both endpoints of the edge are expressed in the given tissue. We further connect the top layer and the middle layer by undirected edges that correspond to known associations between diseases and proteins, and we weight these edges by a positive real-valued parameter *α*.

At the bottom layer, given a collection of protein complexes, we connect each of them to all of its member proteins in the PPI network at the middle layer by undirected edges, while leaving protein complexes unconnected. We weight the introduced edges by a positive real-valued parameter *β*.

Formally, we describe the disease-disease similarity network by a weight matrix **D** = (*d*_*ij*_)_*l*×*l*_, where *l* is the number of diseases and *d*_*ij*_ is the weight of the edge between the *i*th and *j*th diseases or 0 if the edge is absent. We describe the tissue-specific PPI network by a weight matrix **P** = (*p*_*ij*_)_*m*×*m*_, where *m* is the number of proteins and *p*_*ij*_ is the weight of the edge between the *i*th and *j*th proteins or 0 if the edge is absent. We describe connections between the diseases and proteins by a weight matrix **A** = (*a*_*ij*_)_*l*×*m*_, where *a*_*ij*_ = *α* is the weight of the edge between the *i*th disease and the *j*th proteins or 0 if the edge is absent. We describe connections between proteins and complexes by a weight matrix **B** = (*b*_*ij*_)_*m*×*n*_, where *b*_*ij*_ = *β* is the weight of the edge between the *i*th protein and the *j*th complex or 0 if the edge is absent. Put together, the disease-protein-complex network can be represented using a block matrix, as

(1) $$\begin{array}{c} H=\left(\begin{array}{ccc} D & A & 0\\ A^{T} & P & B\\ 0 & B^{T} & 0 \end{array}\right), \end{array}$$

where 0 stands for a zero matrix and the superscript *T* stands for the transposition of a matrix.

### 2.3. Random Walking on the Disease-Protein-Complex Network

We achieve the goal of identifying protein complexes related to a specific query disease by calculating a score for each candidate complex and then rank the candidates to obtain a ranking list. The higher the rank, the more likely to be related to the query disease. For this purpose, we adapt the random walk with restart model [11, 27] to the constructed disease-protein-complex network.

At a quick glance, our model simulates the process that a random walker wanders on the three-layered disease-protein-complex network. When starting on, the walker chooses the query disease of interest as the starting point. In each step of the walking process, the walker may start on a new journey with probability *γ* or move on with probability 1 − *γ*. When moving on, the walker may move at random to one of its direct neighbours in the same layer, jump from the disease layer to the protein layer or vice versa, or jump from the protein layer to the complex layer or vice versa.

Formally, as illustrated in Algorithm 1, we use a vector **q** ^(0)^ = (*q*_*i*_^(0)^)_(*l*+*m*+*n*)×1_ to represent initial probabilities when a random walker starts a journey, with *q*_*i*_^(0)^  (*i* = 1,…, *l* + *m* + *n*) being the probability that the walker initially starts from the *i*th node. In this vector, the element corresponding to the query disease is set to 1, and all of the other elements are set to 0. We normalize each row of the weight matrix **H** for the disease-protein-complex network to obtain a transition matrix **T** = (*t*_*ij*_)_(*l*+*m*+*n*)×(*l*+*m*+*n*)_, in which *t*_*ij*_ = *h*_*ij*_/∑_*j*=1_^*l*+*m*+*n*^ *h*_*ij*_ represents the probability that a random walker moves from the *i*th node to the *j*th node, with each node being a disease, a protein, or a complex. We use a vector **q** ^(*t*)^ = (*q*_*i*_^(*t*)^)_(*l*+*m*+*n*)×1_ to represent probabilities that the random walker stays on nodes at step *t*, with *q*_*i*_^(*t*)^  (*i* = 1,…, *l* + *m* + *n*) being the probability that the walker stays on the *i*th node. We then have the iterative updating formula as

(2) $$\begin{array}{c} q^{\left(t+1\right)}=\left(1-\gamma \right)T^{T}q^{\left(t\right)}+\gamma q^{\left(0\right)}. \end{array}$$

After a number of updates, the probabilities that the random walker staying on nodes will reach a steady state, which can be determined by checking whether the difference between **q** ^(*t*)^ and **q** ^(*t*+1)^ is sufficiently small. In our implementation, we check whether the *L*_2_ norm of Δ**q** = **q** ^(*t*+1)^ − **q** ^(*t*)^ is less than or equal to a small positive number *ϵ* (with the default value 10^−5^). With the steady-state probability (denoted by **q** ^(*∞*)^) obtained, we further calculate a normalized score *s*_*i*_ for the *i*th complex as

(3) $$\begin{array}{c} s_{i}=\frac{q_{l+m+i}^{\left(\infty \right)}}{\sum_{i=1}^{n}q_{l+m+i}^{\left(\infty \right)}} \end{array}$$

and use this score to quantify the strength of association between the complex and the query disease. With such scores calculated for candidate complexes, we further rank the candidates in nonincreasing order according to their scores to obtain the final ranking list.

In this paper, we set the default values for the parameters as disease-protein weight *α* = 1, protein-complex weight *β* = 1, and restart probability *γ* = 0.5. By simulation studies, we find that our model is not sensitive to these parameters (see results for details).

### 2.4. Validation Method

We adopt a leave-one-out cross-validation experiment to assess the capability of our method to identify protein complexes that are associated with human diseases. For this reason, we define a protein complex as associated with a disease if at least one member protein of the complex has been annotated as associated with the disease, and we collect a set of test protein complexes as those associated with at least one disease. Then, in each validation run, we take a test protein complex, identify a query disease as the one with which the complex is associated, pretend that all annotated associations between the query disease and proteins (or corresponding genes) are unknown, and then rank the test protein complex against a collection of control protein complexes.

In the context of the disease-protein-complex network, the above validation procedure is equivalent to remove all edges connecting the query disease and proteins and see whether protein complexes containing these proteins could receive high ranks. In the context of genetics, this validation procedure is equivalent to hide all known genetic bases of the query disease and see whether some of them could be recovered at the protein complex level.

### 2.5. Evaluation Criteria

We adopt three classes of criteria to quantify the performance of our method. First, let us suppose that we have performed a total of *N* validation runs and collected the same number of ranking lists. We calculate a criterion named TOP which is the number of test protein complexes ranked first in their corresponding list. We also divide this number by *N* to obtain the fraction of first ranked test protein complexes and call this fraction precision (PRE). Second, we calculate the average rank of all test protein complexes as the second criterion called mean rank (MR). Alternatively, we normalize ranks of test protein complexes by the lengths of ranking lists to obtain relative ranks, and we calculate the average relative rank of all test protein complexes to obtain mean relative rank (MRR). Third, given a threshold of the relative rank, we calculate the sensitivity (true positive rate) as the fraction of test protein complexes ranked above the threshold and the specificity (true negative rate) as the fraction of control protein complexes ranked below the threshold. Varying the threshold value from 0.0 to 1.0, we draw a rank receiver operating characteristic (ROC) curve and further calculate the area under this curve (AUC). Obviously, larger TOP (PRE)/AUC and smaller MR/MRR indicate higher performance.

## 3. Results

### 3.1. Data Sources

We obtained disease-tissue associations from the literature [28]. Briefly, Lage et al. studied co-occurrence patterns of disease-tissue pairs in PubMed abstracts and quantified the strength of association between a disease and a tissue by a normalized Ochiai's coefficient [29], resulting in a matrix that contains association scores between 926 diseases and 60 tissues. Following the literature [18], we associated a disease with the tissue of the highest score among all tissues, obtaining a total of 926 disease-tissue associations.

We obtained disease-disease similarity scores from the literature [23]. Briefly, van Driel et al. used terms in the anatomy and disease sections of the medical subject headings vocabulary (MeSH) [30] as a standard vocabulary to analyse the full-text and clinical synopsis fields of OMIM records. By characterizing a disease using a vector composed of weighted phenotypic terms, they quantified the similarity between two diseases as the cosine of the angle of their vectors and obtained a matrix that contains pairwise similarity scores for 5,080 diseases [23].

We obtained tissue-specific PPI networks from the literature [18]. Given a specific tissue and a generic PPI network (9,998 proteins as nodes and 41,049 interactions as edges) extracted from the Human Protein Reference Database (HPRD) [24], Magger et al. derived two tissue-specific PPI networks for each of the 60 tissues by using both the edge reweight strategy and the node removal strategy [18].

We extracted disease-protein associations from the Ensembl database using the tool Biomart [31], obtaining a total of 5,164 associations between 3,504 diseases and 3,066 proteins (on February 26, 2013). Focusing on diseases with similarity scores and proteins that can be mapped back to the HPRD database, we obtain 1,962 associations between 1,548 diseases and 1,244 proteins.

We extracted 1,343 human protein complexes from the core set of the CORUM database (release in February 2013) [26], each of which contains at least one protein that can be mapped back to the HPRD database. By considering a protein complex as associated with a disease if at least one of its member protein has been annotated as associated with the disease, we collected a set of 939 disease-related protein complexes as test cases.

### 3.2. Performance of the Proposed Method

With the collected data and the default parameter setting (*k* = 15, *α* = 1, *β* = 1, *γ* = 0.5), we constructed a disease-protein-complex network that was composed of 5,080 diseases, 9,998 proteins, and 1,343 protein complexes. There were a total of 107,661 edges in the network, among which 58,448 are between diseases, 41,049 are between proteins, 1,962 are connecting diseases and proteins, and 6,202 are connecting proteins and protein complexes.

**Figure 2:**
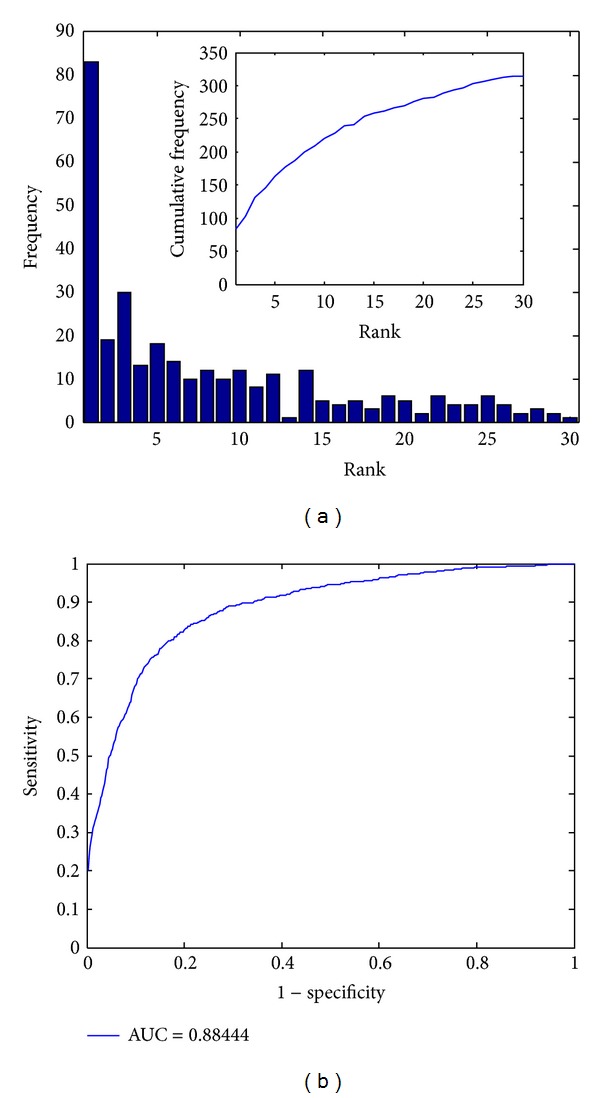
Performance of the proposed method. (a) Histogram of the ranks for the test protein complexes in the validation experiment. (b) The rank receiver operating characteristic (ROC) curve.

We then performed the leave-one-out cross-validation experiment using this network and showed the results in Figure 2. By counting the number of test protein complexes with different ranking position, we observed that 83 (8.84%) test cases were ranked first, 163 (17.36%) were ranked among top 5, 221 (23.54%) were ranked among top 10, and 281 (29.93%) were ranked among top 20. In contrast, a random guess procedure that assigns ranks to protein complexes at random was only expected to rank 0.70 (0.07%) test cases at first (939/1343 ≈ 0.7, 1/1343 ≈ 0.07%), 3.50 (0.37%) among top 5, 6.99 (0.74%) among top 10, and 13.98 (1.49%) among top 20. These results, as illustrated in Figure 2(a), therefore strongly suggest the effectiveness of our method in identifying disease-related protein complexes from a collection of candidates.

We further calculated the proposed evaluation criteria in Algorithm 1 and plotted the ROC curve in Figure 2(b). According to these results, our method achieves a TOP (PRE) of 83 (8.84%), a mean rank (mean relative rank) of 169.04 (12.59%), and an AUC of 88.44%, also supporting the effectiveness of this approach. The ROC curve, as shown in Figure 2(b), climbs fast towards the top-left corner of the plot and again suggests the effectiveness of our method.

A naïve thinking of identifying disease-related protein complex is to quantify the strength of associations between proteins and the query disease and then sum over the scores of member proteins to obtain a score for a protein complex. The main difference between this naïve approach and our method is that when a protein is contained in multiple protein complexes, the score of the protein will be counted multiple times (once for a protein complex) in the naïve approach, while with our method, such phenomenon will not happen because the probability of going out from the protein will be distributed uniformly to the multiple protein complexes in the random walk procedure. We performed a comparison between these two methods and showed the results in Table 1. It is clear, according to this table, that our approach outperforms the naïve approach in all of the three criteria. In detail, our method achieves a TOP of 83, a mean rank of about 169.04, and an AUC of 88.44%, while the naïve approach obtains these criteria as 75, 180.49, and 87.57%, respectively, all supporting the conclusion that our method performs better than the naïve approach.

### 3.3. Comparison of Different Strategies for Constructing the Disease Similarity Layer

**Figure 3:**
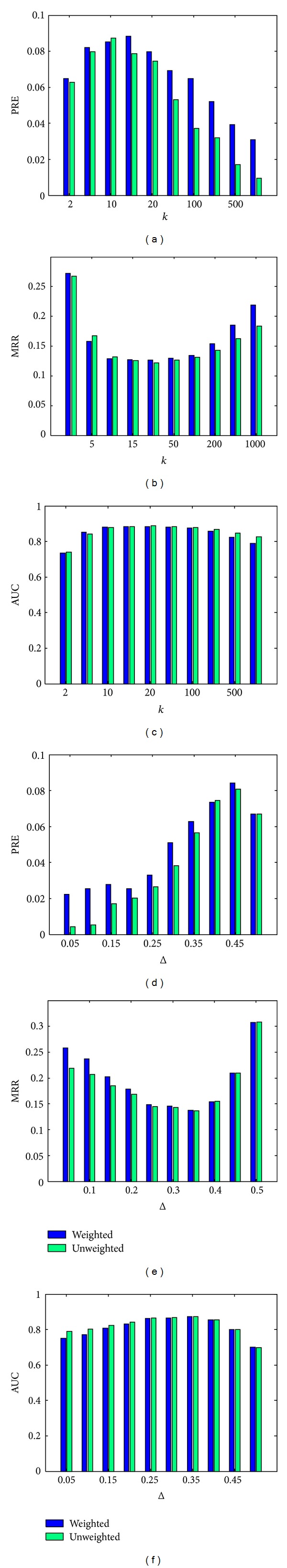
Comparison of different strategies for constructing the disease similarity layer. ((a)–(c)) PRE, MRR, and AUC for the *k*-NN strategy. ((d)–(f)) PRE, MRR, and AUC for the *δ*-threshold strategy.

We considered two strategies for constructing the disease similarity network at the top layer of the disease-protein-complex network: the *k*-nearest neighbour (*k*-NN) strategy and the *δ*-threshold strategy. In both strategies, we further considered two variations: weighting edges by the original similarity values or treating edges as unweighted. We then conducted a comparative study of these strategies and presented the results in Figure 3.

We first observe that our method with the weighted disease similarity network outperforms that with the unweighted one in terms of the precision of test protein complexes (PRE), and the difference between these two variations is subtle according to the other two criteria (MRR and AUC), though the weighted one slightly outperforms the unweighted one. For example, with the *k*-NN strategy and the default parameter setting, the PRE, MRR, and AUC are 8.84%, 12.59%, and 88.44% for the weighted variation, respectively, and 7.88%, 12.71%, and 88.32% for the unweighted one, respectively. Using the *δ*-threshold strategy (*δ* = 0.35) with the default parameter setting, the PRE, MRR, and AUC are 6.28%, 13.69%, and 87.34% for the weighted variation, respectively, and 5.64%, 13.72%, and 87.30% for the unweighted one, respectively. With these observations, we conjecture that the weighted disease similarity network is preferred by our method and will use this network as the top layer of our disease-protein-complex network in the rest of this paper.

Second, we also observe that our method is quite robust to the number of neighboring diseases in the *k*-NN strategy. All of the three criteria only show small fluctuations in a wide range of the parameter *k*. Focusing on weighted networks, the PRE, MRR, and AUC are in general greater than 3.94% 16.25%, and 84.76%, respectively, when *k* is greater than 10 and less than 500, with the optimum values of these criteria achieved at *k* = 15, 20, and 20, respectively. For the *δ*-threshold strategy, our method is also quite robust when the cut-off value *δ* is not too large. Also focusing on weighted networks, the PRE, MRR, and AUC are in general greater than 3.30%, 20.97%, and 79.99%, respectively, when *δ* is greater than 0.25 and less than 0.45, with the optimum values of these criteria achieved at *δ* = 0.45, 0.35, and 0.35, respectively. With these observations, we conclude that the selection of the parameters *k* and *δ* is not critical and kind of flexible. To achieve a balance over all of the three criteria, we recommend to select *k* = 15 and *δ* = 0.35 as default values of these parameters.

Third, we notice that the *k*-NN strategy gives us higher performance than the *δ*-threshold does in a wide range of parameter settings. When comparing the performance at the default parameters, the PRE, MRR, and AUC are 8.84%, 12.59%, and 88.44%, respectively, for the *k*-NN strategy and 6.28%, 13.69%, and 87.34%, respectively, for the *δ*-threshold strategy. Around these parameters, the *k*-NN strategy exhibits consistent higher performance than the *δ*-threshold strategy in terms of both MRR and AUC. Therefore, we recommend the use of the *k*-NN strategy in the construction of the disease similarity network.

### 3.4. Comparison of Different Strategies for Constructing the Protein-Protein Interaction Layer

We considered two strategies to construct the tissue-specific PPI network at the middle layer of the disease-protein-complex network: the node removal strategy and the edge reweight strategy. Besides, we also considered the use of a tissue-nonspecific PPI network extracted from the HPRD database as the middle layer. We then performed a comparison study of these strategies and presented the results in Table 2.

We first observe from this table that the difference between the node removal strategy and the edge reweight strategy is subtle. For example, with the default parameter setting, the PRE, MRR, and AUC are 8.84%, 12.55%, and 88.49% for the node removal strategy, respectively, and 8.84%, 12.59%, and 88.44% for the edge reweight strategy, respectively. This observation is consistent with a previous study about relying on a tissue-specific PPI network to prioritize candidate genes [18]. Therefore, following the literature [18], we focus on the edge reweight strategy in our study because the network constructed using this strategy exhibits preferred properties in connectivity.

We then notice from Table 2 that the tissue-specific PPI network gives us a better performance than the tissue-nonspecific one. For example, with the default parameter setting, the PRE, MRR, and AUC are 8.84%, 12.59%, and 88.44% for the tissue-specific PPI with edge removal strategy, respectively, and 7.99%, 13.97%, and 87.03% for the tissue-nonspecific one, respectively. Therefore, we use the tissue-specific PPI network as the middle level of our disease-protein-complex network.

### 3.5. Robustness to the Parameters Involved

**Figure 4:**
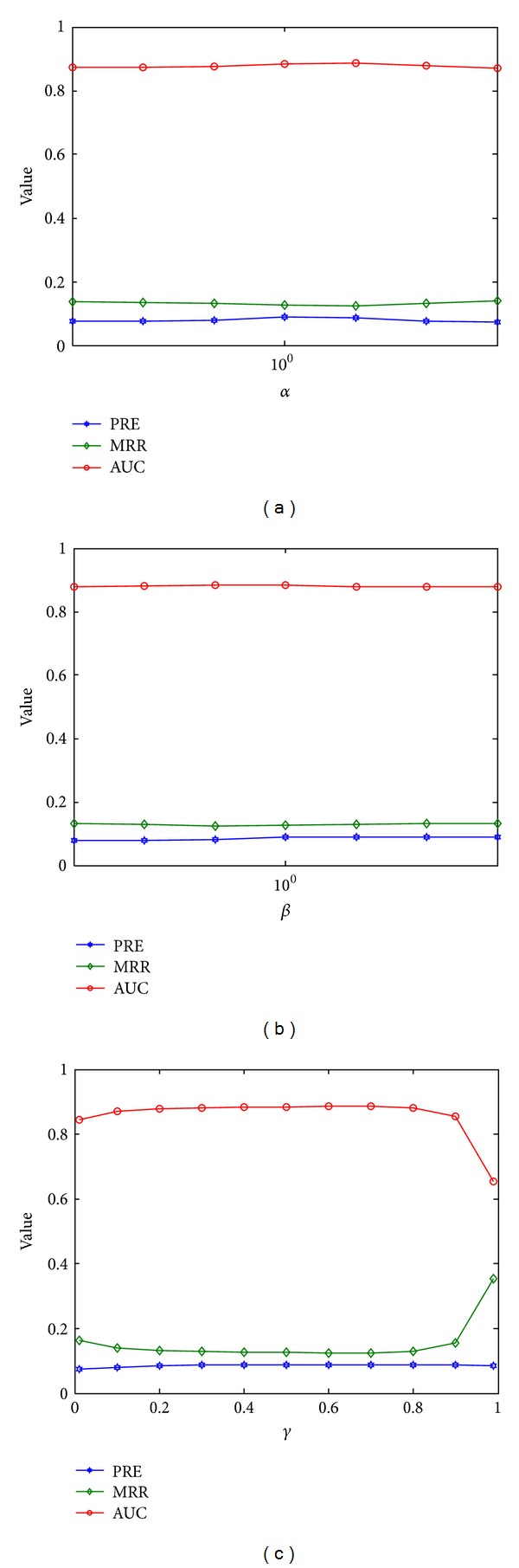
Influence of the parameters involved. (a) Influence of the weights of the disease-protein connections (*α*). (b) Influence of the weights of the protein-complex connections (*β*). (c) Influence of the restart probability (*γ*).

There are three main parameters involved in our method: the weights of the disease-protein connections (*α*), the weights of the protein-complex connections (*β*), and the restart probability in the random walk model (*γ*). To study the influence of these parameters on our method, we performed a comparative study on different values of these parameters and presented the results in Figure 4.

The weights of the disease-protein connections (*α*) determine the possibility of jumping from the disease layer to the protein layer and vice versa. With a large value of *α*, it is easier to travel between the two layers, while with a small value of *α*, it is harder to travel between the two layers. From Figure 4(a), we observe that our method is quite robust to this parameter. In a wide range of this parameter (10^−3^ to 10^3^), all of the three criteria show only tiny fluctuations. For example, at the lower end of the spectrum (*α* = 10^−3^), the PRE, MRR, and AUC are 7.56%, 13.64%, and 87.38%, respectively, while at the higher end of the spectrum (*α* = 10^3^), the PRE, MRR, and AUC are 7.24%, 14.07%, and 86.95%, respectively. Moreover, at the optimum point (*α* = 1), the PRE, MRR, and AUC are 8.84%, 12.59%, and 88.44%, respectively. From these observations, we conjecture that the selection of this parameter is not critical to the performance of our method. We hence use *α* = 1 as the default value for this parameter.

Similarly, the weights of the protein-complex connections (*β*) determine the possibility of jumping from the protein layer to the complex layer and vice versa. With a large value of *β*, it is easier to travel between the two layers, while with a small value of *β*, it is harder to travel between the two layers. From Figure 4(b), we observe that our method is also quite robust regarding this parameter. In a wide range of this parameter (10^−3^ to 10^3^), all of the three criteria show only tiny fluctuations. For example, at one end of the spectrum (*β* = 10^−3^), the PRE, MRR, and AUC are 7.88%, 13.29%, and 87.74%, respectively, while at the other end of the spectrum (*β* = 10^3^), the PRE, MRR, and AUC are 8.84%, 13.26%, and 87.76%, respectively. Moreover, at the optimum point (*β* = 10), the PRE, MRR, and AUC are 9.05%, 13.05%, and 87.94%, respectively. From these observations, we conclude that the selection of this parameter is not critical to the performance of our method. Therefore, we use *β* = 1 as the default value for this parameter.

The restart probability (*γ*) determines the possibility of jumping from any node in the network back to the starting point of the query disease. With a large value of *γ*, a random walker cannot go far away from the starting point and thus will mainly explore neighbouring nodes of this point, while with a small value of *γ*, the random walker is able to explore areas far away from the starting query disease. From Figure 4(c), we observe that our method is robust regarding this parameter, except for extreme values. In a wide range of this parameter (0.1 to 0.8), all of the three criteria show only tiny fluctuations. For example, at one end of the spectrum (*γ* = 0.1), the PRE, MRR, and AUC are 8.09%, 13.92%, and 87.1%, respectively, while at the other end of the spectrum (*γ* = 0.8), the PRE, MRR, and AUC are 8.73%, 12.86%, and 88.18%, respectively. At the optimal point (*γ* = 0.6), the PRE, MRR, and AUC are 8.63%, 12.49%, and 88.54% respectively. Moreover, at the middle point of the spectrum (*γ* = 0.5), the PRE, MRR, and AUC are 8.84%, 12.59%, and 88.44%, respectively, not very different from the optimum point. From these observations, we conclude that the selection of this parameter is not critical to the performance of our method. Therefore, we seek for the simplicity to select *γ* = 0.5 as the default value for this parameter.

### 3.6. Robustness to the Network Structure

**Figure 5:**
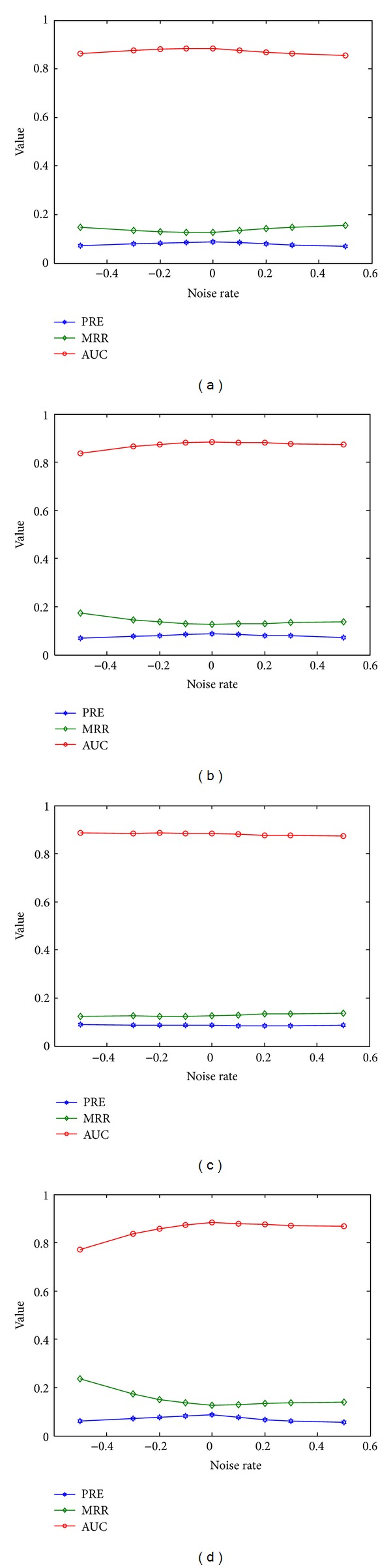
Influence of the addition or removal of edges. Results are the performance of our method with the addition (>0) or removal (<0) of a proportion of edges (a) between diseases, (b) connecting diseases and proteins, (c) between proteins, and (d) connecting proteins and protein complexes. All results are average of 5 independent runs.

There are four types of connections in the heterogeneous network: edges between diseases, connecting diseases and proteins, between proteins, and connecting proteins and protein complexes. These connections determine the structure of the disease-protein-complex network. We then studied how the performance of our method changed with the addition or removal of a proportion of edges and presented the results in Figure 5.

From the figure, we see that our method is quite robust to the addition of edges. For example, when adding 10% edges between diseases into the network, the PRE, MRR, and AUC change from 8.84%, 12.59%, and 88.44% to 8.39%, 13.43%, and 87.59%, respectively. When adding other types of edges, we observe similar robust pattern. Particularly, the performance of our method is quite robust to the noise in the protein-protein interaction network, because the criteria only change slightly with the addition of this type of edges. These observations suggest the robustness of our method to false positive edges in the network.

Our method is also robust to the removal of edges. For example, when removing 10% edges connecting diseases and proteins from the network, the PRE, MRR, and AUC change from 8.84%, 12.59%, and 88.44% to 8.54%, 12.90%, and 88.14%, respectively. When removing 10% edges connecting proteins and protein complexes from the network, the PRE, MRR, and AUC change to 8.22%, 13.66%, and 87.36%, respectively. Again, the performance of our method is quite robust to the noise in the protein-protein interaction network, because the criteria only change slightly with the removal of this type of edges. These observations suggest that our method is also robust to false negative connections in the network.

### 3.7. Predicted Landscape of Associations between Diseases and Protein Complexes

With the performance and robustness of our method demonstrated, we further applied our method to a total of 926 diseases with tissue association information in our data set and predicted associations between these diseases and a total of 1,343 protein complexes. The lists of diseases, protein complexes, and the predicted score for each pair of disease and protein complexes are available for free downloading at our website http://bioinfo.au.tsinghua.edu.cn/jianglab/complex.

## 4. Conclusions and Discussion

In this paper, we have proposed a method for the identification of protein complexes that are related to a query disease via random walking on a heterogeneous network that is composed of a disease layer, a protein layer, and a protein complex layer. We have shown the high performance of our approach via a large-scale leave-one-out cross-validation experiment and have demonstrated the robustness of our approach to the parameters involved. As an application of our approach, we have predicted a landscape of associations between diseases and protein complexes.

Our method has the following advantages. First, in the disease layer, a disease is connected to its neighboring diseases with similar phenotype properties. Therefore, our method is capable of predicting associations for a query disease whose genetic basis is unknown by borrowing information from its neighboring diseases. Second, our method allows the inclusion of the recent discovery about the tissue specificity of protein-protein interactions, leading to high accuracy in making predictions. Finally, our method shows great robustness to the parameters involved, and hence it is easy to be adapted to the analysis of other data.

Certainly, our method can further be extended from the following directions. First, the disease similarity network plays a key role in our method. Besides the phenotype similarity profile derived from MeSH, there are also alternative profiles derived from the unified medical language system (UMLS) [32] and the human phenotype ontology (HPO) [33]. It has been shown that integrated use of these profiles provides a more comprehensive view of correlations in clinic properties of human diseases [34]. The way to integrate these similarity profiles in our current heterogeneous network will be a direction worth exploring.

Second, although the PPI network provides a systematic view of functional similarities between genes, such genomic information as transcriptional regulation, noncoding RNA regulation, functional annotation, pathway annotation, and structure domain annotation also provides useful assessments on functional similarities between genes. Integrating such genomic information with tissue-specific gene expression data to obtain a more comprehensive characterization of tissue-specific functional similarities between genes and further enhance the performance of our method will be one of our future research directions.

Third, protein complexes represent higher level functional units than proteins. Besides, gene modules such as pathways can be thought of as even higher level function units. Therefore, it also matters to pursue the goal of identifying pathways or gene modules that are associated with a given query disease. In technology, our method can be directly applied to solve this problem.

Finally, the predicted genome-wide landscape of associations between human diseases and protein complexes provides a rich resource in understanding genetic bases of human inherited diseases. Using these prediction results to facilitate the analysis of prevalent genetic data such as single nucleotide polymorphisms identified in traditional genome-wide association studies or recent exome sequencing studies will also be a goal worth pursuing.

## Figures and Tables

**Algorithm 1:**
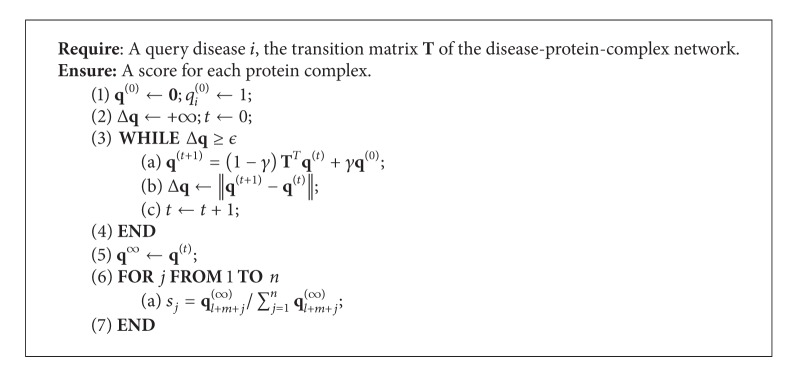
The random walk algorithm on the disease-protein-complex heterogeneous network.

**Table 1:** Comparison of the proposed approach and the naïve approach.

	Proposed method	Naïve approach
Top (PRE)	83 (8.84%)	75 (7.99%)
MR (MRR)	169.04 (12.59%)	180.49 (13.44%)
AUC	88.44%	87.57%

**Table 2:** Comparison of different strategies for constructing the protein-protein interaction network.

	Edge reweight	Node removal	HPRD
TOP (PRE)	83 (8.84)%	83 (8.84)%	75 (7.99)%
MR (MRR)	169.04 (12.59%)	168.52 (12.55%)	187.65 (13.97%)
AUC	88.44%	88.49%	87.03%

## References

[1] Manolio TA (2010). Genomewide association studies and assessment of the risk of disease. *The New England Journal of Medicine*.

[2] The Wellcome Trust Case Control Consortium (2007). Genome-wide association study of 14,000 cases of seven common diseases and 3,000 shared controls. *Nature*.

[3] Choi M, Scholl UI, Ji W (2009). Genetic diagnosis by whole exome capture and massively parallel DNA sequencing. *Proceedings of the National Academy of Sciences of the United States of America*.

[4] Biesecker LG (2010). Exome sequencing makes medical genomics a reality. *Nature Genetics*.

[5] Ehret GB, Munroe PB, Rice KM (2011). Genetic variants in novel pathways influence blood pressure and cardiovascular disease risk. *Nature*.

[6] Bromberg Y (2013). Chapter 15: disease gene prioritization. *PLoS Computational Biology*.

[7] Chen Y, Zhang WS, Gan MX, Jiang R (2012). Constructing human phenome-interactome networks for the prioritization of candidate genes. *Statistics and Its Interface*.

[8] Altshuler D, Daly M, Kruglyak L (2000). Guilt by association. *Nature Genetics*.

[9] Aerts S, Lambrechts D, Maity S (2006). Gene prioritization through genomic data fusion. *Nature Biotechnology*.

[10] Wu X, Jiang R, Zhang MQ, Li S (2008). Network-based global inference of human disease genes. *Molecular Systems Biology*.

[11] Li Y, Patra JC (2010). Genome-wide inferring gene-phenotype relationship by walking on the heterogeneous network. *Bioinformatics*.

[12] Vanunu O, Magger O, Ruppin E, Shlomi T, Sharan R (2010). Associating genes and protein complexes with disease via network propagation. *PLoS Computational Biology*.

[13] Wu X, Liu Q, Jiang R (2009). Align human interactome with phenome to identify causative genes and networks underlying disease families. *Bioinformatics*.

[14] Chen Y, Jiang T, Jiang R (2011). Uncover disease genes by maximizing information flow in the phenome-interactome network. *Bioinformatics*.

[15] Lage K, Karlberg EO, Størling ZM (2007). A human phenome-interactome network of protein complexes implicated in genetic disorders. *Nature Biotechnology*.

[16] Jiang R, Gan MX, He P (2011). Constructing a gene semantic similarity network for the inference of disease genes. *BMC Systems Biology*.

[17] Zhang W, Sun F, Jiang R (2011). Integrating multiple protein-protein interaction networks to prioritize disease genes: a bayesian regression approach. *BMC Bioinformatics*.

[18] Magger O, Waldman YY, Ruppin E, Sharan R (2012). Enhancing the prioritization of disease-causing genes through tissue specific protein interaction networks. *PLoS Computational Biology*.

[19] Guan Y, Gorenshteyn D, Burmeister M (2012). Tissue-specific functional networks for prioritizing phenotype and disease genes. *PLoS Computational Biology*.

[20] Jiang B, Wang J, Xiao J, Wang Y (2009). Gene prioritization for type 2 diabetes in tissue-specific protein interaction networks. *Lecture Notes in Operations Research*.

[21] D’Andrea AD (2003). The fanconi anemia/BRCA signaling pathway: disruption in cisplatin-sensitive ovarian cancers. *Cell Cycle*.

[22] Yang P, Li X, Wu M, Kwoh C-K, Ng S-K (2011). Inferring gene-phenotype associations via global protein complex network propagation. *PLoS ONE*.

[23] van Driel MA, Bruggeman J, Vriend G, Brunner HG, Leunissen JAM (2006). A text-mining analysis of the human phenome. *European Journal of Human Genetics*.

[24] Keshava Prasad TS, Goel R, Kandasamy K (2009). Human protein reference database—2009 update. *Nucleic Acids Research*.

[25] Su AI, Wiltshire T, Batalov S (2004). A gene atlas of the mouse and human protein-encoding transcriptomes. *Proceedings of the National Academy of Sciences of the United States of America*.

[26] Ruepp A, Brauner B, Dunger-Kaltenbach I (2008). CORUM: the comprehensive resource of mammalian protein complexes. *Nucleic Acids Research*.

[27] Köhler S, Bauer S, Horn D, Robinson PN (2008). Walking the interactome for prioritization of candidate disease genes. *American Journal of Human Genetics*.

[28] Lage K, Hansena NT, Karlberg EO (2008). A large-scale analysis of tissue-specific pathology and gene expression of human disease genes and complexes. *Proceedings of the National Academy of Sciences of the United States of America*.

[29] Rentzsch R, Orengo CA (2009). Protein function prediction—the power of multiplicity. *Trends in Biotechnology*.

[30] Lowe HJ, Barnett GO (1994). Understanding and using the Medical Subject Headings (MeSH) vocabulary to perform literature searches. *Journal of the American Medical Association*.

[31] Smedley D, Haider S, Ballester B (2009). BioMart—biological queries made easy. *BMC Genomics*.

[32] Bodenreider O (2004). The Unified Medical Language System (UMLS): integrating biomedical terminology. *Nucleic Acids Research*.

[33] Robinson PN, Köhler S, Bauer S, Seelow D, Horn D, Mundlos S (2008). The human phenotype ontology: a tool for annotating and analyzing human hereditary disease. *American Journal of Human Genetics*.

[34] Gottlieb A, Stein GY, Ruppin E, Sharan R (2011). PREDICT: a method for inferring novel drug indications with application to personalized medicine. *Molecular Systems Biology*.

